# Prenatal identification of partial 3q duplication syndrome

**DOI:** 10.1186/s12920-019-0547-y

**Published:** 2019-06-13

**Authors:** Magdalena Pasińska, Rafał Adamczak, Anna Repczyńska, Ewelina Łazarczyk, Barbara Iskra, Agata Klaudia Runge, Olga Haus

**Affiliations:** 1 Department of Clinical Genetics, Faculty of Medicine, Collegium Medicum in Bydgoszcz, Nicolaus Copernicus University, Skłodowskiej - Curie 9, 85-094 Bydgoszcz, Poland; 2 Prenatal Genetic Clinic, Department of Obstetrics and Gynecology, Faculty of Medicine, Collegium Medicum in Bydgoszcz, Nicolaus Copernicus University, Ujejskiego 75, 85-168 Bydgoszcz, Poland

**Keywords:** Prenatal diagnosis, Ultrasounds, 3q duplication, Genetic syndrome

## Abstract

**Background:**

The 3q duplication syndrome is a result of duplication of a large fragment of the long arm of chromosome 3, mainly 3q21-qter, and in most cases it is diagnosed only after birth. The phenotypic consequences resulting from genetic imbalance are an important source of information for genetic counselling, especially in prenatal diagnostics. However, in most cases it is impossible to define them precisely because the final clinical presentation is a result of an overlap, usually due to different sizes of deletions and/or duplications not only chromosome 3 but also of translocation partner chromosome. In this article, we present a prenatal diagnosis of the 3q duplication syndrome in a foetus, arising from a balanced insertion ins (7,3)(q21.2;q12.3q29) carried by the mother.

**Case presentation:**

The article presents a case of a 29-year-old woman referred to the Genetic Outpatient Clinic for consultation in the 12th week of her fifth pregnancy with a diagnosis of generalised hydrops foetalis. The analysis of karyotype using GTG technique and FISH allowed diagnosis of a balanced aberration in the mother, and determined the type of chromosomal rearrangement, which allowed the identification of the origin of the additional genetic material in the foetus and the previous malformed child of the same couple. The use of molecular karyotyping techniques (FISH and aCGH) allowed a precise determination of the size of the imbalanced fragments in the affected siblings.

**Conclusions:**

The aCGH technique is particularly valuable for the diagnostics of submicroscopic deletions and duplications, if no imbalanced chromosomal aberrations are detected by routine cytogenetic tests. It is also a valuable technique for identifying and fully characterizing genetic material of unknown origin, which can’t be identified using routine cytogenetic techniqes. However, it does not allow identification of balanced aberrations in carriers.

## Background

Chromosomal abnormalities causing genomic imbalance are a major cause of congenital developmental defects and intellectual disability. They arise de novo or as a result of asymmetric segregation of genetic material during meiosis in a gamete of a parent carrying a balanced aberration, usually a reciprocal translocation or inversion. If an asymmetric segregation occurs, the resulting gametes contain a combination of partial trisomy or partial monosomy [1, 2].

The phenotypic consequences resulting from genetic imbalance are an important source of information for genetic counselling, especially in prenatal diagnostics. However, in most cases it is impossible to precisely define them because the final clinical presentation is the result of an overlap, usually due to different sizes of deletions and/or duplications of genetic material [3, 4].

The 3q duplication syndrome is a result of duplication of a large fragment of the long arm of chromosome 3, especially 3q21-qter, and in most cases it is diagnosed only after birth. In patients with this syndrome, the observed defects include abnormalities of the central nervous system, facial dysmorphia, congenital heart defects, defects of the urogenital tract, intellectual disability and growth disturbances [4–6].

The article presents a prenatal diagnosis of the 3q duplication syndrome in a foetus, arising from a balanced insertion ins (7,3)(q21.2;q12.3q29) carried by the mother.

## Case presentation

A 29-year-old woman (II-11) was referred to the Genetic Outpatient Clinic for consultation in the 12th week of her fifth pregnancy with a diagnosis of generalised hydrops foetalis.

In anamnesis, the child - a boy from her fourth pregnancy, born prematurely using Caesarean section at 36 weeks of gestation, with an Apgar score of 7, birth weight 2600 g (> 50th centile), length 47 cm (> 50 centile), occipotofrontal head circumference 34 cm (> 10 centile), had numerous birth defects. They were as follows: heart defects, i.e. ventricular septal defect (VSD) and atrial septal defect II (ASD II); central nervous system (CNS) defects, i.e. lack of the cerebellar vermis and slight enlargement of the lateral ventricles; eyeball defects, i.e. defects of the eyelids and eyeballs, and lenticular staphyloma diagnosed in the left eye.

An orofacial cleft, hearing loss, and limb defects were also present. Among the dysmorphic features observed in the child were: synophrys, eyelids defect, wide nose root, anteverted nostrils, large maxilla, small retracted mandible, dysmorphic auricles.

The child died at 8 months of age due to a complex heart defect with pulmonary hypertension and circulatory insufficiency.

Medical records were obtained after the child’s death, and the previously begun genetic diagnostics was completed only after the diagnosis of congenital defects of the foetus in the subsequent pregnancy (III-22), using earlier sampled material from the child (III-21). The remaining three older children showed no signs of disease.

The mother had a sister with a clinically diagnosed Down syndrome (II-14) and other six healthy siblings who had healthy children. The information about the clinical diagnosis of Down syndrome in the sister was obtained while collecting the interview from the mother before performing prenatal tests. However, the family did not agree to carry out genetic diagnosis in the patient. Therefore, the final diagnosis is unknown.

**Fig. 1:**
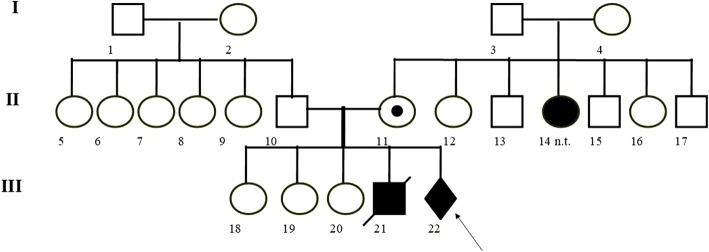
Pedigree of the patient with balanced insertion and karyotype: 46, XX, ins (7;3)(q21.2;q12.3q29). White symbol (square or circle) - a healthy person. A symbol (square or circle) with a black dot - a carrier of the balanced insertion ins(7;3). Black symbol (square or circle) - a person with mental retardation (MR) and congenital malformations. Black diamond - an unbalanced structural aberration in the foetus with congenital malformations. n.t. - a person with MR, not tested genetically. Arrow - proband

In the family of the child’s father (II-10), no congenital defects or disabilities were found (Fig. 1).

In the ultrasound examination done in the fifth pregnancy, the foetal CRL (Crown–Rump Length) value at 12 weeks was 50 mm and NT (Nuchal Translucency) was 8 mm, while in a biochemical examination MoM for PAPP-A was 0.47, and MoM for BHCG was 1.49.

Nuchal translucency [NT] is an ultrasound marker used in non-invasive prenatal screening to assess the risk of foetal aneuploidy in the first trimester of pregnancy. It arises due to the accumulation of fluid around the neck of the foetus. In the examined case, in the foetus the NT was 8 mm (> 95th centile) with enlargement of the skin edema up to the sacral and pericranial regions. The basic mechanism of formation of non-immune hydrops foetalis - (NIHF) are imbalances between the production and absorption of interstitial fluids. Generalized swelling may be a consequence of increased venous pressure in the course of foetal failure, the presence of abnormal structures in the chest, increased capillary permeability, impaired lymphatic flow or reduced plasma oncotic pressure.

A combination of nuchal translucency with the maternal serum PAPP-A and B-hCG has a detection rate of approximately 90% for trisomies 21,18 and 13 with a 5% false positive rate.

Because of the abnormal presentation of the foetus in ultrasound examination and an elevated risk of aneuploidy shown in at 15 weeks of pregnancy, the mother decided to undergo amniocentesis with amniocytes genetic tests.

**Fig. 2:**
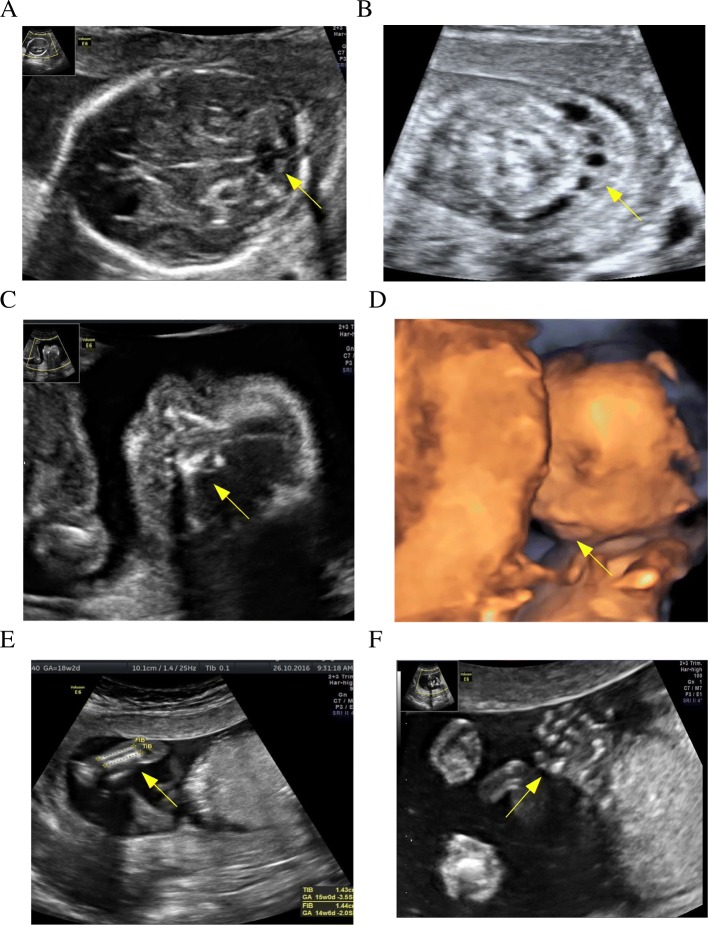
Abnormal foetal image in ultrasound examination at 18 weeks of gestation. **a** - hypoplasia of the cerebellar vermis, absent cavum septum pellucidum, **b** - cystic hygroma, **c** - hypertelorism, abnormal nostrils, broad nasal root, **d** - retrognathia, **e** - shortening of the long bones, **f** - clinodactyly of the 5th finger

In the ultrasound examination at 18 weeks of pregnancy, intrauterine growth restriction (IUGR) of 2 weeks was found. Furthermore, abnormal foetal head contour, with agenesis of the corpus callosum, enlargement of the lateral ventricle anterior horns and hypoplasia of the cerebellar vermis, were diagnosed. The nuchal fold thickness (NF) of 8.5 mm with numerous cysts was found. Abnormal facial profile with retrognathy, hypertelorism and anteverted nostrils was also noted. The palatopharyngeal arch had an extended angle with preserved continuity (Fig. 2). In the spine, important shortening of the sacrocaudal section, and in the limbs shortening of all long bones by approximately 2 weeks in relation to other dimensions of foetal biometry and its age, were observed. Hands with a normal number of fingers and bilateral clinodactyly of the fifth finger were found. Moreover, subaortic ventricular septal defect (VSD), double outlet right ventricle (DORV) and pericardial effusion were observed.

The parents (II - 10 and II-11) were informed about the unfavourable prognosis due to severe brain abnormalities and other foetal defects, high probability of its death in utero or shortly after birth, and if surviving, severe intellectual disability. Therefore, the parents elected a termination of the pregnancy.

## Methods

The study material for determining foetal karyotype was amniotic fluid sampled by amniocentesis. Amniocytes, obtained by centrifugation of amniotic fluid, were cultured for two weeks in the complete media AmnioMax from Gibco and Amnio Grow Plus from Cytogen. For the analysis of the constitutional karyotype of the parents and the brother with developmental defects (using earlier sampled material - a suspension of cultured and fixed cells), peripheral blood (PB) was used. PB lymphocytes were cultured for 72 h with medium RPMI-1649 Medium Sigma-Aldrich, supplemented with LF-7 mitogen. The culture was carried out and terminated in routine conditions. Cytogenetic preparations were stained using the GTG technique, karyotypes were established and further analysed using fluorescence in situ hybridisation (FISH). Chromosome painting probes were used to label chromosomes 3 and 7: Chromosome 3 Whole Chromosome Painting Probe from Cytocell, Chromosome 7 Whole Chromosome Painting Probe from Cytocell, and ToTelVysion MIX 3 from Vysis. The FISH analysis was carried out according to the protocols of manufacturers.

Array comparative genomic hybridisation (array-CGH) was carried out using a commercially available array (CytoSure, Constitutional v3 (8x60k), Oxford Gene Technology (OGT), Oxfordshire, UK), according to the manufacturer’s protocol. The reported nucleotide coordinates were based on UCSC Genome Browser on Human Feb. 2009 (GRCh37/hg19) Assembly. The CytoSure Interpret Software (OGT) was used for genomic copy-number analysis.

## Results

**Fig. 3:**
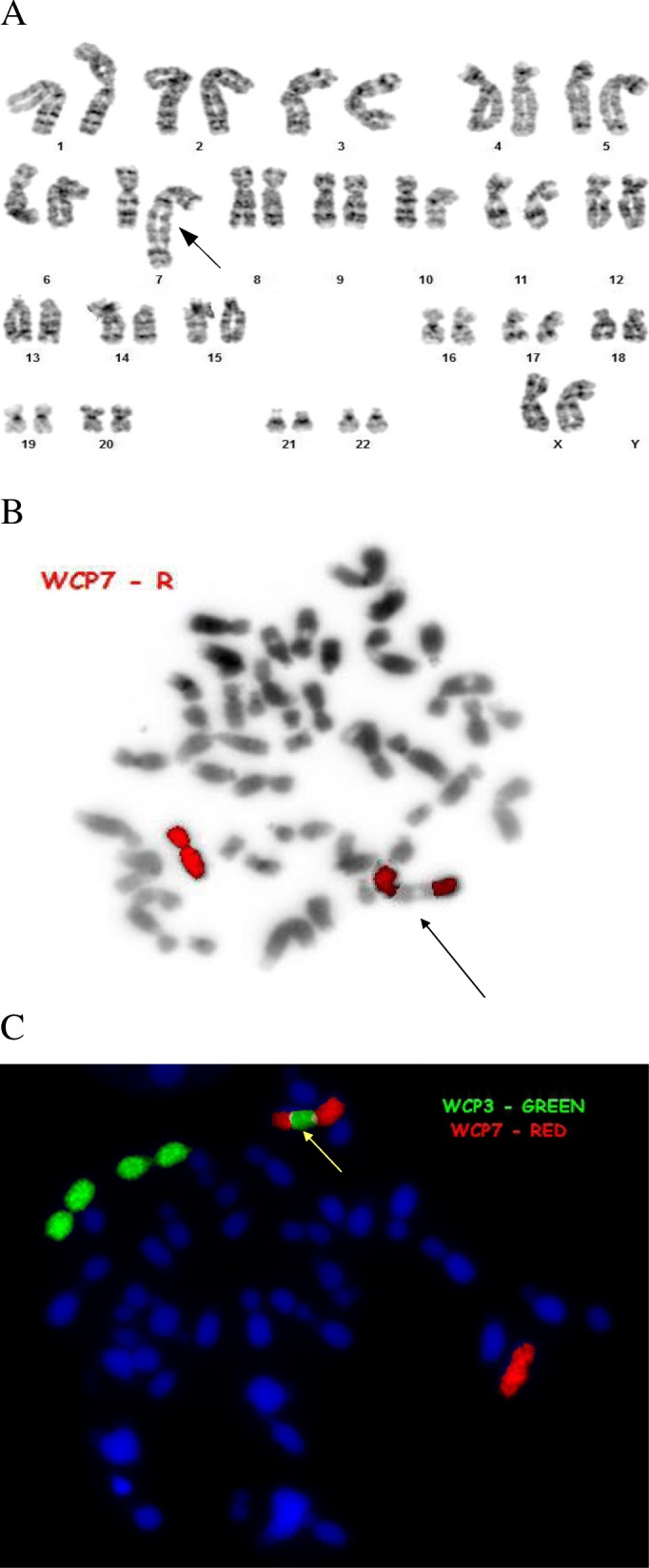
**a**. Karyogram of the foetus (patient III/22) in GTG-banding showing. 46,XX,der(7)ins(7;3)(q21.2;q12.3q29)mat. The arrow indicates abnormal chromosome 7. **b**. Metaphase spread stained using FISH with a whole-chromosome painting probe for chromosome 7 in fetus III/22. Additional material does not derive from chromosome 7. The arrow indicates abnormal chromosome 7 with additional material of unknown origin. **c**. Metaphase spread stained by FISH with whole chromosome painting probes: 7 - red, 3 - green in patient III/22. Additional material from chromosome 3 is present on the long arm of chromosome 7. Arrow indicates abnormal chromosome 7 with additional material of chromosome 3

Additional genetic material of unknown origin was detected on chromosome 7 of the foetus in a GTG examination. FISH analysis allowed exclusion of duplication of a fragment of chromosome 7 (Fig. 3a and b). Abnormal karyotype with an insertion of unknown genetic material into chromosome 7 was established: 46,XX,der(7)ins(7;?)(q21.2;q?).

**Fig. 4:**
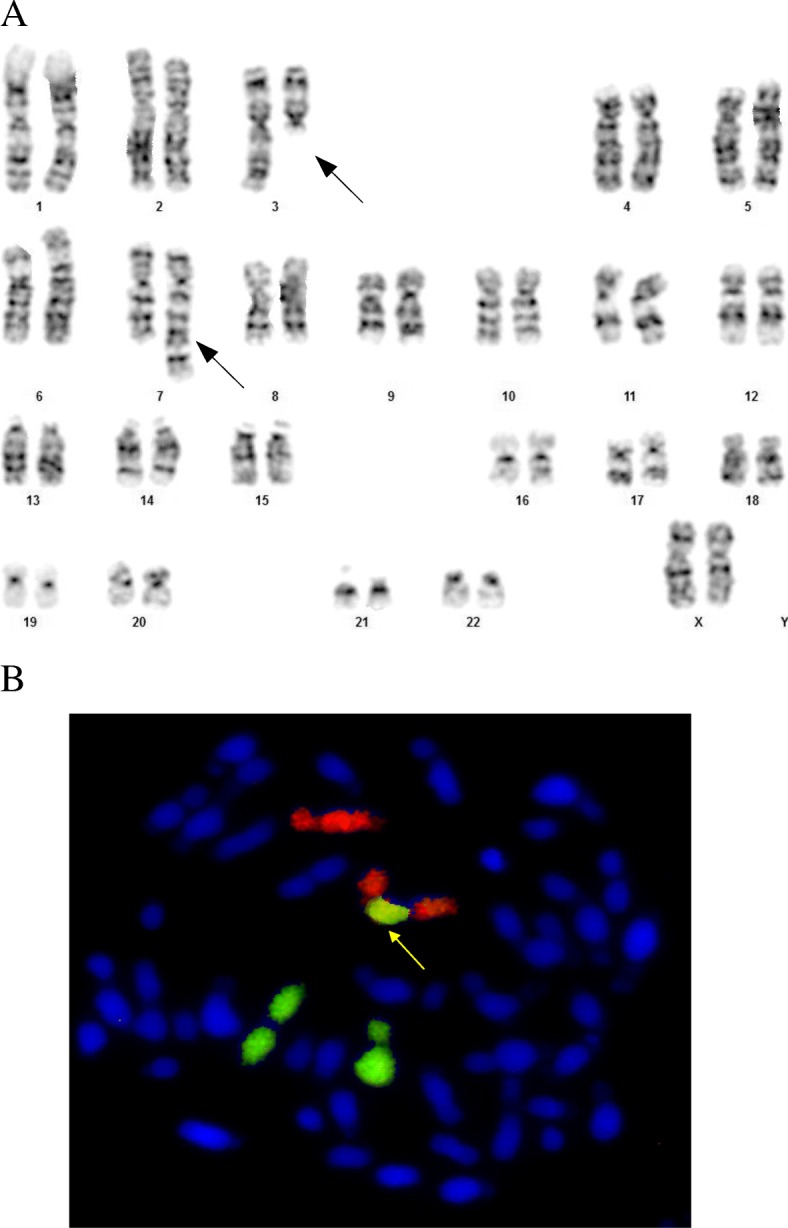
**a**. Karyogram of the mother (patient II/11) stained using GTG-banding, showing 46,XX,ins(7;3)(q21.2;q12.3q29). The arrows indicate abnormal chromosomes 3 and 7, with the inserted fragment from chromosome 3. **b**. Metaphase spread of FISH with whole-chromosome painting probes: 7 - red, 3 - green. Additional material from chromosome 3 is present on the long arm of chromosome 7. The arrow indicates abnormal chromosome 7 with the inserted fragment from chromosome 3

To elucidate the character of the aberration, cytogenetic tests were conducted in both parents. The mother was diagnosed with a carrier status for a balanced insertion of chromosome 3 into chromosome 7: 46,XX,ins(7;3)(q21.2;q12.3q29) (Fig. 4a and b). This allowed identification of the additional genetic material in the foetus, the final karyotype of which was: 46,XX,der(7)ins(7;3)(q21.2;q12.3q29)mat (Fig. 3c).

In the previously established constitutional karyotype of the patient’s deceased older child, all analysed metaphase spreads contained a chromosome 7 derivative with an unknown insertion: 46,XY,der(7)ins(7;?)(q22;?). In the FISH analysis of this material, the following chromosome painting probes were used: 2, 4, 5, 6, 8, 10 and 13, as well as a probe specific for C-MYC (8q22.21), and it was found that the additional material did not originate from any of these chromosomes.

Identification of the asymptomatic carrier status in the mother allowed identification of the origin of the additional genetic material in the karyotype of this child, in whom the same aberration as in the diagnosed foetus was found. A post-mortem molecular test using a whole-genome oligonucleotide microarray (based on the GRCh37 Assembly) with a mean resolution of 60 kbp showed a duplication of 91.72 Mbp, of chromosome 3 material: 3q12.3-q29 (chr3:102998201_194717279), which allowed the ultimate determination of chromosomal breakpoints, with the final karyotype of the deceased child being 46,XY,der(7)ins(7;3)(q21.2;q12.3q29)mat. The aCGH did not show any 7q deletion. This result was not available during the prenatal diagnostics in the subsequent pregnancy.

## Discussion & conclusions

Duplication syndrome involving a large part of chromosome 3 with bands 3q21-qter and 3q25-qter was characterised by Kondo et al. (1979), Garcia-Esquivel et al. (1987) and Montero et al. (1988) [4, 6, 7]. It is a rare genetic syndrome in which many defects are observed, usually diagnosed after birth [7, 8]. The critical region responsible for the typical phenotype of the dup(3q) syndrome has been mapped at the regions 3q26.3–q27.7 [9, 10]. In the cases previously described, patients with the dup(3q) syndrome were diagnosed with brain abnormalities which led to severe mental disability with a tendency for epileptic seizures [1, 11]. The duplicated chromosomal fragment (3q26.3-q27.7) reported by Aqua et al. contains many genes, e.g., *NLGN1, BCHE, TNIK, SOX2* and *Map6D1*, characterized by a high expression during the embryonic development of the brain [9, 10]. The characteristic dysmorphic features, previously described by other authors, such as synophrys, wide nose root, anteverted nostrils, large maxilla and small retracted mandible, were also found in the diagnosed foetus and its ill brother, in whom structural abnormalities of eyeballs and eyelids were also observed [1, 7, 11]. In the examined foetus, the duplicated fragment of the chromosome was larger and included a more proximal region, from 3q21.2. Similarly, Wilson et al. described a patient with corneal opacities and extended optic nerves, as well as underdeveloped olfactory bulbs, while Preiksaitiene et al. described a patient with optic nerve dysplasia [5, 12]. Preiksaitiene et al., based on tests carried out in a family with recurrent syndromic spina bifida due to a familial balanced chromosomal rearrangement, concluded that the spinal defect was caused by a duplication of the 3q21-qter region, and thus covered the proximal region of the chromosome [12, 13]. In the described foetus, a caudal agenesis and significant shortening the long bones of the limbs was observed. Dworschak et al. described a patient with a clinical diagnosis of Curriano syndrome (characterized by a triad of sacral anomalies, anus developmental defects and presacral mass), which was caused by de novo duplicated 3q26.32-q27.2. In addition, the authors showed in a comprehensive review of the dup (3q) literature several patients with caudal defects and caudal anomalies, suggesting that these defects are the innate phenotype of the dup (3q) syndrome [6]. In the examined case III-22, sacral agenesis was diagnosed without presacral teratoma. Due to the fact that the pregnancy was completed in the 18th week, with a foetal weight of about 160 g, a section test confirming the presence of other defects was not performed.

In the literature, omphalocele has also been described in association with the 3q duplication [12, 14]. The phenotypic discrepancy between the patients can be explained by variations of the number of active genes present in chromosomal fragments of different sizes [3, 9, 11]. Numerous developmental defects are the cause of short survival, and according to data from the literature, almost 40% of children with this syndrome die during the first year of life [5, 8, 11].

Although it is critical to study parental karyotypes to help determine recurrence risk and the origin of the unknown chromosomal material, this also highlights the value of using a microarray for cases with unknown additional genetic material. This is particularly true if the rearrangement is de novo, where studying parental karyotypes does not help with identification. Precise identification of the additional material is also critical for accurate genetic counselling. The inability to carry out tests in parents during the diagnosis of an earlier patient child made it difficult to identify additional genetic material and to make a definitive diagnosis of the deceased child as well as of the foetus.

In the study by Golder et al., only in 1 of 4 cases was a de novo duplication present [15]. Observations made by Gonzales et al. showed that 60–70% of cases of the 3q duplication resulted from a balanced parental translocation, carried mainly by the mother [1]. Similarly, Chih-Ping Chen et al. reported that most cases of the 3q duplication occurred as a result of segregation of parental rearrangements. In as many as 3 out of 4 cases, the duplication arose from parental rearrangements including a pericentric inversion of chromosome 3 [16, 17].

Carriers of balanced chromosomal aberrations, such as those in the tested family are at risk of formation of imbalanced gametes during meiosis as a result of various forms of segregation, which may cause infertility, non-implantation of the embryo, shorter embryo or foetus survival, as well as congenital defects and developmental disorders in children after birth [1, 11, 15]. The analysis of karyotype and FISH allowed diagnosis of a balanced aberration in the mother, and determined the type of chromosomal rearrangement, which allowed identification of the origin of the additional genetic material in the foetus and the previous malformed child of the same couple. The use of molecular karyotyping techniques (FISH and aCGH) permitted a precise determination of the size of the imbalanced fragments in the affected siblings.

Partial chromosome 3q duplication syndrome [dup(3q)] is a well-described condition that has overlapping features with Brachmann de Lange syndrome [4, 9]. The phenotypic manifestations include dysmorphism, microcephaly, synophrys, broad nasal bridge, hirsutism, congenital heart defect, genitourinary disorders, and mental retardation.

The described case of foetal diagnosis is very important, because there are barely a few cases of pure 3q duplication diagnosed prenatally, and the diagnosed foetal abnormalities contribute to the definition of phenotype associated with this condition.

Pure duplications of 3q are rare because most of the reported patients are found to carry unbalanced translocations [6, 8].

The described case is important because a aCGH has not shown any 7q deletions in the unbalanced karyotype. Thus, it represents a case of a pure 3q duplication.

Genetic analysis in children with congenital defects is important for establishing the diagnosis and predicting their further development. The extent of genetic imbalance correlates with the severity of the phenotype. Therefore, patients with a high degree of genetic imbalance have severe developmental defects and abnormalities of psychomotor development, along with high mortality rate [3, 7, 11].

The aCGH technique is particularly valuable for the diagnosis of submicroscopic deletions and duplications, if no imbalanced chromosomal aberrations are detected by routine cytogenetic tests. It is also a valuable technique for identifying and fully characterizing genetic material of unknown origin, which can’t be identified using routine cytogenetic techniques. However, it does not allow identification of balanced aberrations in carriers [1, 5, 6].

## Data Availability

All data generated or analyzed during this study are included in this published article [and its supplementary information files].

## Abbreviations

aCGH: array Comparative Genomic Hybridization
BHCG: free Beta-subunit of Human Chorionic Gonadotropin
CNS: Central Nervous System
CRL: Crown–Rump Length
FISH: Fluorescence in situ Hybridization
GTG: GTG technique
IUGR: Intrauterine Growth Restriction
MoM: Multiple of the Medians
NF: Nuchal Fold Thickness
NT: Nuchal Translucency
PAPP-A: Pregnancy-Associated Plasma Protein-A
PGD: Preimplantation Genetic Diagnosis
